# Diabetic Neuropathic Pain: Directions for Exploring Treatments

**DOI:** 10.3390/biomedicines12030589

**Published:** 2024-03-06

**Authors:** Yuchen Chen, Xue-Jun Song

**Affiliations:** 1 Faculty of Medicine, Imperial College London, London SW7 2AZ, UK; yuchen.chen22@imperial.ac.uk; 2 Department of Medical Neuroscience and SUSTech Center for Pain Medicine, Southern University of Science and Technology, Shenzhen 518055, China

**Keywords:** diabetic neuropathy, diabetic neuropathic pain, medication, non-pharmacological interventions, targeted treatments

## Abstract

Diabetic neuropathic pain (DNP) is one of the common and severe late-stage complications of diabetes mellitus, which could greatly influence the patients’ quality of life. Patients with DNP often experience spontaneous pain and evoked pain such as mechanical allodynia and thermal hyperalgesia, meaning that their physical and psychological health are severely impaired. Unfortunately, the mechanisms of DNP remain highly elusive, so substantial breakthrough in effective DNP targeted treatments is still clinically challenging. This article will hence summarise the main mechanisms currently known to underlie DNP pathogenesis, along with describing some of the current and potential treatment methods against diabetic neuropathic pain.

## 1. Introduction

Millions of people all around the world nowadays are suffering from diabetes. Diabetic neuropathic pain (DNP), one of the most common complications that affects around 25–50% of patients, is still a major intractable burden [1]. This complication also poses significant challenges for healthcare professionals and researchers. Whilst many researchers have attempted to unveil a series of potential causes, mechanisms, and treatments underlying DNP, unluckily, most of the findings have failed to be brought into clinical practice [2]. In addition, the diagnostic standard of DNP is not universal [3], making this situation even more complicated, leading to delays in care and/or treatment. This article will therefore briefly summarise the possible causes of DNP and some common characteristics of diabetic neuropathy, in addition to the up-to-date and potential related treatments against DNP, based on recent research findings and studies.

## 2. Mechanisms Underlying Diabetic Neuropathic Pain

### 2.1. Hyperglycaemia

**Figure 1:**
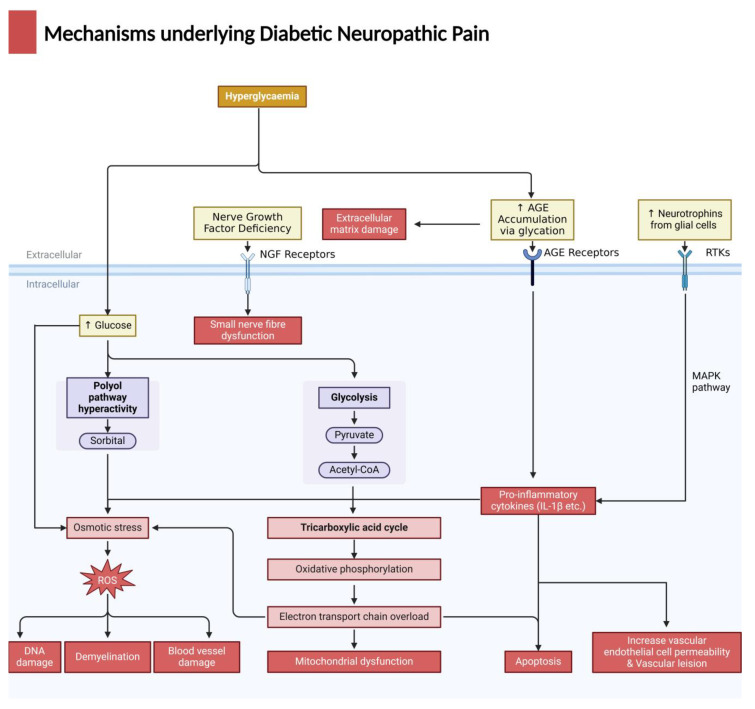
Summary of the proposed mechanisms underlying diabetic neuropathic pain (DNP). Created in Biorender.com.

Diabetic patients suffer from hyperglycaemia (a high blood glucose level) [4] due to their body’s inability to regulate and maintain a normal blood glucose concentration. At the same time, high osmotic pressure and other pathways attributed to long-term hyperglycaemia are highly associated with DNP, through damage to patients’ neurons and decreases in neurovascular flow. There are several well-acknowledged pathways triggered by hyperglycaemia, which scientists believe could lead to DNP (summarised in Figure 1 below).

#### 2.1.1. Polyol Pathway Hyperactivity

The hyper-activation of the polyol pathway, or aldose reductase pathway, contributes to the development of DNP. In this pathway, excessive glucose is converted to sorbitol and then oxidised to fructose with the help of the enzymes called aldose reductase and sorbitol dehydrogenase, respectively [1]. These products are known as “polyols” due to the existence of multiple hydroxyl groups within their structures, and the accumulation of polyols could be consequently detrimental. Firstly, aldose reductase will oxidise NADPH to NADP+ in order to facilitate the conversion of glucose into sorbitol [5]. However, the NADPH is of paramount importance in oxidising glutathione disulfide (GSSG) into glutathione (GSH). The depletion of NADPH could result in a decrease in the concentration of GSH in cells, which gives rise to less hydrogen peroxide being converted into water and the accumulation of reactive oxygen species (ROS). Consequently, cells from the nervous system and the blood vessels will have less resistance to oxidative stresses and will experience attacks from the ROS: their functions will be impaired; and demyelination and cell death within neurons will occur, causing DNP.

#### 2.1.2. Advanced Glycation End Products (AGEs) Accumulation and Myelination Loss

As a result of continuous hyperglycaemia and insufficient enzyme involvement, excessive glucose will also irreversibly react with proteins or lipids to form advanced glycation end products (AGEs) via glycation reactions. The continuous accumulation of AGEs inside tissues such as the blood vessels, kidneys or neurons will give rise to a number of problems [6]. For example, the AGE receptors could bind to the excessive AGEs, triggering the downstream effects in which reactive oxygen species (ROS) will be formed: these damage tissues and neurons (demyelination), resulting in DNP [7]. The nuclear factor κB (NF-κB), which is a type of transcription factor for controlling the expression of pro-inflammatory genes, will be activated as well, leading to the formation of proinflammatory cytokines (e.g., tumour necrosis factor (TNF)-α and interleukin (IL)-1β). These cytokines are found to be strongly associated with inflammation and DNP pathogenesis [1]: The inflammation triggered, especially in neurons, could eventually result in the loss of myelination in neurons and cell apoptosis. Moreover, AGEs could interact with both intracellular proteins and extracellular matrix proteins, modifying them and making them dysfunctional [1].

#### 2.1.3. Inflammation

The body’s immune system is designed to tag potentially foreign and damaging stimuli, either intrinsically or extrinsically. However, Pop-Busui et al. [8] also claimed that inflammatory responses triggered by the immune system could also contribute to the pathogenesis of DNP. An example is that the expressions of specific pro-inflammatory cytokines or adhesion molecules such as IL-1, -6, -18, TNF-α, ICAM-1, VCAM-1 are increasingly elevated in patients with diabetic neuropathy to induce inflammatory responses. Consequently, the permeability of vascular endothelial cells would increase dramatically, leading to potential microvascular complications and further exacerbations of DNP [9], which could be another influencing factor for DNP pathogenesis. Importantly, interactions between glial cells and neurons plays a paramount role in this inflammation-induced neuropathic pain. Glial cells could release neurotrophins (to neurons in the spinal cord) which exacerbate neuronal excitability and pain sensitivity [10]. Signalling pathways such as the MAPK and NF-κB pathways are triggered during this process; these pathways induce the transcription of genes associated with inflammatory responses and neuropathic pain.

#### 2.1.4. Mitochondrial Oxidant Stress

Excessive metabolites could enter mitochondria due to reduced mitochondrial membrane potential caused by a high glucose level, leading to mitochondrial dysfunction. This could result in the electron transport chain in the inner mitochondrial membrane being hyperactivated. Reactive oxygen species would then be generated excessively and, likewise, exert oxidative stress on mitochondria and could directly damage mitochondrial DNA, proteins, and lipids [1,11]. In addition, due to insufficient oxygen supply, pyruvates would be converted into lactic acid (anaerobic respiration). Once lactic acid accumulates in a large amount and NAD+, a vital coenzyme, is depleted, glycolysis will eventually be inhibited, and neuronal function will also be impaired due to lack of energy production [11].

#### 2.1.5. Growth Factor Deficiency

Various growth factors such as insulin-like growth factor (IGF), nerve growth factor (NGF) and neurotrophin 3 (NT3) were found to be related to diabetic neuropathy: disruptions in their levels, especially NGF, could lead to disrupted regeneration of neurons and Schwann cells [12]. NGF is important in the development and maintenance of small-diameter sensory nerve fibres. In patients with diabetic neuropathy, many exhibit NGF deficiency [13]. The deficiency in NGF potentially result in the dysfunction of small nerve fibres. Hence, pathogenesis of diabetic neuropathy would be reinforced. In rat models, the roles of NGF are further confirmed: treating diabetic rats with insulin normalised these deficits, and treatment with exogenous NGF contributes to dose-dependent increases in neuropeptide levels. As a result, these findings indicates that growth factors deficiency could contribute to the diabetic neuropathy, and elevating their level could possibly alleviate or reverse the pathologies [14].

#### 2.1.6. Vascular Lesions

As well as damage to neurons and Schwann cells, lesions of blood vessels and capillaries contribute to the pathogenesis of DNP [6,11]. For example, as mentioned, the accumulation of AGE due to hyperglycaemia could cause capillary proliferation, thicken the basement membrane, and increase capillary permeability, giving rise to ruptures in blood vessels and microangiopathy. On top of that, the protein kinase C, a group of phospholipid-dependent serine/threonine kinases which play a critical role in signal transduction, could be activated because of the buildup of ROS. This might cause several signal transduction pathways being altered, releasing a series of pro-inflammatory factors like IL-1β and TNF-α. As a result, diabetic patients experience diabetic microangiopathy, in which blood carrying oxygen cannot be effectively delivered to the nervous system, causing ischemia, hypoxia, thrombus and lesions of nerve tissues, and eventually induction and maintenance of DNP [1,6,11].

There are many other different pathways, such as immune dysfunctions [15], that are found to be activated by long-lasting hyperglycaemia due to diabetes, giving rise to DNP, but overall, most of them still are elusive to scientists. Moreover, an analysis of the EURODIAB Prospective Complications Study data in June 2023 [16] discovered that females could be more prone to developing diabetic neuropathic pain compared to males (73% painful DNP cases were female compared to 48% painless DNP cases were female, *p* = 0.003), suggesting another risk factor in terms of sex. In terms of symptoms, in general, patients with DNP often experience severe pain, distal muscle numbness, thermal hyperalgesia and mechanical allodynia [1].

Whilst further investigation is needed, studies have revealed the roles of several proteins and their signalling pathways in the pathogenesis of DNP. These will be discussed in detail along with the following treatment method in this article.

## 3. Treatments against DNP

So far, scientists have been trying to find specific treatments against DNP. However, few of them have been proven to be successful or sufficiently curative. Most of the existing treatments are only aimed at relieving patients’ pain, and what makes the situation worse is that a majority of patients cannot respond to some therapies effectively [17], further increasing the difficulties of finding simple and definite solutions. A series of medications and potential targeted treatments will be introduced in the following paragraphs.

### 3.1. FDA-Approved Medicine

A traditional method of treating pain relief is undoubtedly through medication, such as antidepressants for modulating pain signals, anticonvulsants for suppressing abnormal nerve firings, etc. The U.S. Food and Drug Administration (FDA) has currently approved only four drugs for DNP treatment [18]: Tapentadol (an opioid), Pregabalin (an anticonvulsant), Duloxetine (an antidepressant) as well as Capsaicin (a topical analgesic). Specifically, Tapentadol, a μ-opioid receptor agonist, could bind to the μ-opioid receptors in spinal cords to reduce the perception of pain. In addition, it also acts a noradrenaline re-uptake inhibitor [19]. Therefore, noradrenaline concentration in the synaptic cleft could be increased, further activating its descending inhibitory pain pathways. Pregabalin is a type of antiepileptic medication which could be used to treat epilepsy as well as neuropathic pain [20]. It mainly functions by binding to α2δ-1 proteins, which are subunits of voltage-gated Ca^2+^ channels in CNS, limiting the Ca^2+^ influx during pain signalling. Duloxetine acts similarly to the tapentadol as a serotonin–norepinephrine re-uptake inhibitor (SNRI). Serotonin and norepinephrine are neurotransmitters that could dampen the transmission of pain signals to the brain; hence, increasing their concentration helps to alleviate DNP [21]. Capsaicin is often found in chili peppers, whereas it can also be used to relieve DNP. It binds with TRPV1 receptors (responsible for heat sensation) and desensitise these receptors over repeated treatment, so that the patients would gain tolerance towards capsaicin [22].

As stated above, these drugs could only relieve a small amount of patients’ pain. On top of that, a recent multi-centre, randomised, double-blind crossover trial on patients with diabetic peripheral neuropathic pain carried out in UK at 13 centres showed that adverse events such as dizziness, nausea and dry mouth happened after administration of these medicine, and the severities of these adverse effects increased accordingly with the combination of different medication treatments [23]. Therefore, alternative approaches for DNP treatment with less side effects need to be discovered and have recently been emphasised. Recently, eliapixant, a P2 × 3 antagonist (P2 × 3 are ligand-gated cation channels which contribute to the pathogenesis of DNP when increased in expression), entered a randomised, placebo controlled-phase 2a PUCCINI study. However, the results unfortunately failed to illustrate significant differences between changes in pain scores of DNP patients receiving eliapixant vs. those receiving placebo (average of 0.60 in favour of placebo) [24], which accentuated the difficulties in advancement in treatments for painful diabetic neuropathy.

### 3.2. Natural Isolates

A recent study discovered the neuroprotective potential of several natural compounds: berbamine, bergapten, and carveol, on STZ (Streptozotocin)-induced diabetic rats with DNP [25]. Compared with artificially synthesised medicine, natural isolates are believed to generate less side effects and to be more readily accessible. These compounds derived from plants have been long acknowledged for their anti-inflammatory potentials, which is the reason why this study chose to investigate their possible influences on DNP. It was illustrated in this study that, after day 28 of STZ treatment (65 mg/kg, i.p.), the thermal and mechanical thresholds of the DNP rats’ hind paws against external stimuli were significantly increased by treatments with berbamine (5 and 15 mg/kg), bergapten (50 and 100 mg/kg) and carveol (10 and 20 mL/kg) separately. Similar results were shown by the standard treatment group of pregabalin (30 mg/kg). In addition, after applying different assays to investigate the activities of various factors associated with DNP, such as GSH, NF-κB, pro-inflammatory cytokines (TNF-α and Cyclooxygenase 2/COX-2), the results showed that all of the elevated expressions that led to inflammation and diabetic neuropathy due to STZ treatments were significantly lowered by the berbanmine, bergapten and carveol treatments, in comparison with those of STZ-induced diabetic rats with DNP. As a result, these findings further proved the anti-inflammatory effects of these natural compounds and suggested their possible roles in alleviating DNP, which provided a novel therapeutic option other than synthetic medications like pregabalin.

### 3.3. Vitamins

Vitamins are organic compounds which are involved in the regulation of multiple enzymes associated with nerve and bone functions, in addition to homeostasis. What is more, nearly all vitamin (A, B, C, D, E, K) deficiencies were found to be risk factors for developing diabetic neuropathy [26]. The most emphasised were vitamin B and D.

Vitamin B (VB). VB, a group of water-soluble nutrients essential for many metabolic pathways [27], has been studied frequently among scientists in relation to controlling DNP. Reports had shown that VB could be effective against several chronic painful conditions, including DNP [28,29,30,31,32]. One recent study mainly focused on the effects of systematic administration of VBC containing B1, B6, and B12 on STZ-induced rats with DNP by measuring the behavioural and biochemical alterations of P2 × 3 and TRPV1 expression [33]. It was shown that, among the STZ-induced diabetic rats with DNP, daily administration of VBC (B1/B6/B12 = 100/100/2 mg/kg, i.p.) for 7 consecutive days (day 36–42 after STZ injection) significantly attenuated the thermal hyperalgesia and mechanical allodynia when compared to those without VBC treatments. However, this effect was not long-lasting, as it rapidly disappeared after treatment termination. Furthermore, in comparison to the control group, the expressions of P2X3 and TRPV1 in the dorsal root ganglion neurons were significantly higher in STZ-induced hyperglycaemia and DNP rats, as demonstrated by Western blot and immunohistochemistry, whilst these expressions were significantly suppressed by the repeated administration of VBC for 7 days (same dose and method as above), albeit to varying degrees. These results demonstrated that long-term VBC treatment could be potentially feasible in controlling and relieving DNP and this analgesic effect might be mediated by inhibiting the increased expression of P2X3 and TRPV1 (as explained in the above medication section), and both may play important roles in DNP pathogenesis (P2X3: ligand-gated ion channel that transduces nociceptor activation). Nevertheless, according to a systematic review [27], vitamin B treatments still lack strong evidence for their analgesic and neuroprotective benefits. As a result, more clinical trials with higher quality are required in this field of study.

Vitamin D. Vitamin D deficiency was also discovered to be related to peripheral neuropathy triggered by diabetes [34]. Studies have determined that through modulating a variety of receptors and pathways such as Wnt-10α signalling (Wingless-Type MMTV Integration Site Family, Member 10A) and NRF-1 (Nuclear Respiratory Factor 1), the supplementation of Vitamin D could eventually alleviate inflammation and decrease the rate of demyelination of neurons, which are critical factors in DNP [35]. This study found that these therapeutic effects of VD treatment could be attributable to a reduction in cytokines such as TNF-α, IL-18 (pro-inflammatory cytokines), as well as caspase-3 (executor of apoptosis) apoptotic activity. Meanwhile, Bcl-2 and Smad-7 level in the sciatic nerve was augmented, whilst the expressions of NICD1, Wnt-10α, and β-catenin decreased. Mitochondrial biogenesis and function were augmented, as well. This study hence provided a new avenue for the treatment of DNP.

In conclusion, diabetic neuropathy is associated with a range of different types of vitamins, as explained above. And although their specific functions and effects on DNP still need to be further studied, they offer scientists new ideas for exploring DNP treatments.

### 3.4. Specific Enzyme/Receptor Targets

The explorations in potential targeted treatments based on specific receptor and enzyme targets related to DNP are also part of the recent research focus, in which the most significant focus has been on the gelatinases MMP-9 and MMP-2, and the ephrinB–EphB receptors.

#### 3.4.1. MMP-9/2

MMP stands for matrix metalloproteinases, which are a family of enzymes responsible for the breakdown of extracellular matrix (ECM). Neurons and glial cells inside the nervous system also possess this type of enzyme. It is suggested that MMPs could be responsible for different types of pain, such as neuropathic pain and complex regional pain syndrome (CRPS) [36]. Specifically for diabetic patients, they are activated by proinflammatory cytokines like IL-1β, resulting in a further decrease in insulin level in diabetic patients [37]. In combination with cytokines, AGEs, etc., as mentioned in the above mechanism section, MMPs could trigger further loss of functional neurons and DNP through their function of cleaving and breaking down ECM around neuronal cells. Studies showed that two specific types of MMP—MMP-9 and MMP-2—could be responsible for the development of neuropathic pain due to peripheral nerve injury [38] and opiate withdrawal-induced pain enhancement [39]. Another study proved the role of MMP-9 and MMP-2 in DNP pathogenesis in a rat model [40]. It showed that 59.42% of STZ-treated (70 mg/kg i.p.) rats developed DNP, which manifested as mechanical allodynia. MMP-9i, an MMP-9 inhibitor, injected intrathecally at a dose of 10 μg/20 μL to the treatment groups, resulted in significant alleviation of the mechanical allodynia. On the other hand, MMP-2 acted as a negative regulator, because while the activity of MMP-9 was enhanced in DNP rats, that of MMP-2 was reduced in the dorsal root ganglion (DRG) and spinal cord. Moreover, the roles of MMP-2, which were distinct from MMP-9, were further confirmed following the mitigation of mechanical allodynia after administration of MMP-2 into DNP rats. The behavioural analysis, Western blot and immunohistochemistry carried out illustrated that MMP-9 expression was increased in the DRG and dorsal horn (DH) of the DNP rats’ spinal cord. Administration of MMP-9i was able to limit the increase in expressions of proinflammatory cytokines, microglia and astrocytes, which are highly associated with DNP pathogenesis (the specific functions of astrocytes were discussed in a later study [41]: astrocytes were found to release these cytokines, and inhibition of motor cortex astrocytes could lead to relief in DNP among STZ-induced diabetic models). In contrast, MMP-2 expression was significantly decreased among the STZ-induced DNP models. The roles of MMP-9 in DNP were further proved in MMP-9 double knock-out mice: MMP-9^−/−^ mice [40]. Firstly, out of 80 MMP-9^−/−^ mice injected with STZ, around 52–100% of them showed significantly alleviated mechanical allodynia compared to wild type STZ-induced DNP mice. According to electron micrographs taken, in addition to myelin basic protein markers (MBP) and myelin degraded markers (DMBP) analysis from the sciatic nerve and DH after STZ treatment, wild type STZ-induced DNP mice exhibited myelin abnormalities and axonal degeneration, whereas these conditions were not shown and were significantly reversed in MMP-9^−/−^ mice. In consequence, this study discovered that in STZ-induced DNP models, the MMP-9 expression increased, which could lead to myelin abnormalities, especially in the peripheral nerves. Contrarily, MMP-2 served as a negative regulator for DNP pathogenesis. Targeted mutations and inhibitions of MMP-9 could be a new research direction for DNP attenuation.

#### 3.4.2. EphrinB–EphB Receptor Signals

EphB1 receptor is one of the fourteen members of the largest subfamily of mammalian receptor tyrosine kinases (RTKs)-Eph (erythropoietin-producing human hepatocellular) receptors. Previous studies discovered that ephrinB–EphB receptor signalling pathway is highly associated with the development and maintenance of chronic pain due to a number of factors such as bone cancers and nerve injuries [42,43,44,45]. In summary, ephrinB–EphB signalling contributes to the onset of central sensitization. This is one of the underlying mechanisms of many types of chronic pain, and it is mediated via NMDA receptors leading to synaptic plasticity. The roles of EphB1 in DNP were investigated in STZ- and alloxan- induced diabetic rats with DNP [46]. Contrary to what was shown following nerve injuries [42,46], Western blot analysis demonstrated that only the phosphorylated form of EphB1 (pEphB1) expressions, not both of the EphB1 and pEphB1, increased significantly in the spinal cords of DNP rats, which could suggest a different mechanism of DNP pathogenesis from other types of peripheral nerve injuries. In addition, the targeted blocking of EphB1 by EphB1-Fc (5 μg per dose, i.t.) could only significantly alleviate the already-established mechanical allodynia in rats in late phases (day 26 after STZ treatment), not in early phases, with the effects of a single dose lasting for 4 h. The effectiveness of EphB1 blocking was also further confirmed by the following Western blot and immunohistochemistry analysis, which showed that the elevated expressions of the astrocytes, IL-1β and TNF-α (highly associated with DNP) were suppressed significantly following spinal blocking EphB1 receptors. These findings implied that rather than assisting the induction of DNP, EphB1 receptor signalling in the spinal cord seemed to be more related to maintaining the currently established DNP in diabetic models.

#### 3.4.3. Multi-Chemokine Receptor Antagonist: RAP-103

A recent study investigated the roles of RAP-103, a multi-chemokine receptor (CCR2/CCR5/CCR8) antagonist, in relieving pain caused by diabetic peripheral neuropathy [47]. It was previously found out that these chemokine receptors, when bound to their corresponding chemokines, are involved in monocytes recruitment and microglia activation, releasing pro-inflammatory factors and increasing the excitability of nociceptive neurons. These, in turn, induce inflammation and pain [48]. Thus, this research studied the effect of the receptor antagonist peptide RAP-103 on inhibiting DNP in STZ-induced diabetic rat models, after gaining inspiration from a previous study on RAP-103 [49]. Ruff et al. [47] discovered that the STZ-treated (60 mg/kg) diabetic rat models with DNP established mechanical and cold allodynia, whereas these situations were significantly reversed after daily oral gavage of RAP-103: a dose of as small as 0.02 mg/kg per day was found to be effective. Furthermore, the administration of RAP-103 seemed to have a better and more significant effect on reversing mechanical allodynia, because it only partially reversed cold hypersensitivity among the DNP rats. Followed by a further qPCR analysis on the mRNA expressions of inflammatory markers in the sciatic nerves of DNP rat models, the results suggested that administration of RAP-103 was also able to significantly decrease the previously established high expressions of the chemokine CCL3 and the cytokines IL-1β and TNF-α because of the diabetes induced by STZ administration. However, it showed fewer reducing effects on CCL2 and CCR5 levels; meanwhile, it did not change the expression of CCR2. Consequently, this study found some potential effects of RAP-103 in alleviating DNP and controlling its related inflammatory biomarkers.

There are also other promising target receptors for attenuating DNP, such as 5-HT1A [50], that have yet to be investigated further. In conclusion, the targeted treatments based on the specific enzyme and receptor signalling mentioned above related to DNP could be investigated further in future studies with the aim of translating the findings into clinical significance.

### 3.5. Gut Microbiota

Numerous microbes are located in the gastrointestinal (GI) tract, and besides their regular functions such as assisting digestion and GI tract motility, studies have found that they are also involved in regulating various physiological processes and immune functions [51] as well as the development of various types of neuropathic pain [52,53,54]. One proposed mechanism is that the lipopolysaccharides (LPS) released from the gut microbiota could contribute to the production of pro-inflammatory factors by macrophages, such as IL-1β. These factors are highly related to the sensitisation of peripheral pain receptors [55]. Recently, it has been reported that gut microbiota play important roles in DNP, as well as in neuropathic pain induced by other tissue damage [56]. Studies showed that gut microbiota depletion alone resulting from a combination of four different antibiotics (ABX) containing vancomycin (0.5 g/L), ampicillin (1 g/L), neomycin (1 g/L) and metronidazole (1 g/L) did not change the thermal and mechanical sensitivity in mice. However, there is a potential relationship between gut microbiota depletion and DNP pathogenesis in STZ-induced (i.p., 40 mg/kg for 5 consecutive days) diabetic mice. A 2-week-long pre-treatment with ABX before the STZ treatments successfully prevented an increase in blood glucose level in mice, as well as the following mechanical allodynia or thermal hyperalgesia, whereas ABX treatment administered simultaneously to STZ treatment only limited the increase in blood glucose concentration to a small extent. In addition, although it successfully alleviated the mechanical allodynia, the administration of ABX 42 days after STZ treatment failed to prevent the increase in blood glucose level. These results suggest that the pre-treatment of ABX to cause gut microbiota depletion could be the preferrable method to prevent the induction of STZ-induced diabetes, and that gut microbiota might be involved in the induction, but not maintenance of a high blood glucose level due to STZ-induced diabetes. On top of that, the induction and maintenance of mechanical allodynia both require the existence of gut microbiota. In the following analysis, the study attempted to confirm the roles of gut microbiota by transplanting faecal bacteria from regular specific-pathogen-free (SPF) mice to mice receiving a 2-week-long ABX treatment prior to STZ administration, via daily oral gavage (one dose for 3 consecutive days at day 35 after STZ treatment). The following faecal analysis confirmed the ability of the transplantation to rescue gut microbiota depletion, and the transplantation rapidly and fully recovered the mechanical allodynia which was previously prevented by ABX pre-treatment. Meanwhile, among all phyla of bacteria, the Akkermansia, Bacteroides, and Desulfovibrionaceae phyla could be essential because they were positively correlated with the restoration of gut microbiota and the induction of painful behaviours.

### 3.6. Non-Pharmacological Approaches

A series of non-pharmacological approaches for treating DNP were discovered, often in combination with conventional treatments for achieving optimal pain-relieving effects [57]. For example, spinal cord stimulation (SCS) was found to relieve chronic neuropathic pain via activating opioid receptors, reducing the sensitivity of hyperexcited neurons in dorsal horns [58]. Hence, through implantation of electrodes [59], the effects of SCS on relieving painful diabetic neuropathy were investigated by two randomised controlled clinical trials published in 2014 [57,59]. The first one [60] was a multi-centre clinical trial studying the effects of SCS on 36 DNP patients who did not respond well to conventional treatments over a 6-month period, among which only 59% of patients treated by SCS showed improvements. It is worth mentioning that one death case was reported in one of the DNP patients receiving SCS due to subdural hematoma after a dural puncture. The second trial [61] was based on an open randomised parallel-group design: 60 DNP patients were randomised in a 2:1 proportion (the best conventional medical approach in addition to SCS treatment (SCS group): alone without SCS (control group)). The investigation lasted for 6 months, and significant reductions in pain intensity (around 60%) were displayed in 93% (37/40) of the SCS-treated DNP patients compared to the control group. However, side effects occurred, as diabetic patients were likely to be more susceptible to infections and the resulting complications, especially during the implantation of the pulse generator for SCS.

Moreover, a common and key procedure recorded in traditional Chinese medicine, acupuncture, is often used for pain relief. This procedure is mainly based on inserting tiny needles into specific points called “acupoints” on one’s body. Tang et al. [62] suggested that acupuncture could involve purinergic signalling, which is essential to the sensation and transmission of pain signals in the periphery towards the CNS. Thus, its potential effects on painful diabetic neuropathy have also been studied by scientists. The findings in patients with DNP were fully satisfactory: a single-blind, placebo-controlled RCT carried out by Garrow et al. recruited 45 DNP patients [63]. The results showed that acupuncture as an additional treatment slightly improved the pain intensity among DNP patients, whilst the improvements were not statistically significant compared to those in the sham group. However, unlike spinal cord stimulation, what was meaningful in this study was that acupuncture demonstrated no noticeable side effects. In addition, a recent study by Qu et al. [64] investigated the effects of electroacupuncture (EA) on STZ-induced diabetic rat models, which showed that EA could alleviate the hyperalgesia of DNP rats, possibly by limiting the elevated expression of microglial P2X4R.

Exercise has also been found to be possibly beneficial in relieving DNP [65]. In addition to conventional drug therapy, exercise therapy with less significant side effects has also been introduced by scientists. Hence, its effects on improving diabetic neuropathic pain have been widely studied. In summary, pro-inflammatory factors associated with DNP pathogenesis such as IL-1β, TNF-α, IL-6, IL-10, etc., were discovered to be down-regulated by different forms of exercises in DNP patient models (resistance and peripheral neuropathy exercise) or rat models (swimming exercise) [66,67]. The downregulation of these factors could contribute to the dampening of DNP and associated inflammation. Furthermore, in patients with DNP, integrated or aerobic/aerobic and resistance exercises all resulted in lowered HbA1c levels (indicator for blood glucose concentration) [68,69,70]. Thus, these studies implied that patients with DNP, by performing proper exercises, could have a better internal glucose level control. Treadmill exercise in rat models with DNP was also found to be beneficial in relieving DNP by increasing NO synthesis [71] and regulating apoptosis [72], etc.

Overall, these findings demonstrated the promising effects of non-pharmacological therapies against painful diabetic neuropathy, albeit to varying degrees. It is of paramount importance for more and larger (especially multinational) randomised trials to be carried out in order to assess the feasibility of applying different non-pharmacological interventions against DNP into clinical practice worldwide (either individually or as supplementations to traditional therapies). In addition, the new-rising nanotechnology (e.g., nanoscale materials such as nanoparticles which can be integrated with drugs to attenuate internal oxidative stress) also highlighted its usefulness for targeting DRG in alleviating diabetic neuropathic pain [73,74].

## 4. Conclusions

The mechanisms and treatments underlying DNP have long been investigated, whereas many existing discoveries are still limited and have failed to achieve clinical significance. This review summarises the main mechanisms related to DNP development, as well as its existing and potential therapeutical options (summarised in Table 1), such as FDA-approved medications; treatments that target specific molecules; and non-pharmacological interventions, each with their own pathways and side effects. In general, understanding and elucidating the exact process of DNP pathogenesis is of paramount importance for developing strategies against DNP. The abovementioned findings related to DNP could possibly direct scientists by providing a series of potential paths for controlling and alleviating DNP. More investigations and evaluations should be conducted to achieve a deeper understanding of the practicability of each potential treatment.

## Figures and Tables

**Table 1:** Summary information of existing/potential therapeutic methods for DNP.

Treatments/Targets	Signalling Pathway/Molecules Involved	Side Effects	Reference
Tapentadol *	Activation of μ-opioid receptor and inhibition of noradrenaline re-uptake	Nausea, sleepiness, constipation, headache	[19]
Pregabalin *	Binding of α2δ-1 (voltage-gated Ca_2+_ channels); decrease in Ca_2+_ influx	Drowsiness, swelling hands and feet, sleepiness, headache	[20]
Duloxetine *	Increase in conc. of serotonin and norepinephrine in synaptic cleft; dampening of pain signal transmissions	Drowsiness, nausea, constipation, increased sweating.	[21]
Capsaicin *	De-sensitisation of TRPV1; tolerance of capsaicin	Nausea, dry mouth, difficulty breathing	[22]
Natural Isolates (e.g., berbamine, bergapten, carveol)	Lowered expression of GSH, NF-κB, cytokines (TNF-α and COX-2)	Nausea (Rare)	[25]
Vitamin B	Inhibition of increased expression of P2X3 and TRPV1	N/A	[27,28,29,30,31,32,33]
Vitamin D	Wnt-10α signalling + NRF-1. Reduction of TNF-α and IL-18, Caspase-3 activity	N/A	[34,35]
MMP-9/2 (Enzymes)	TNF-α, IL-1β. Cleavage of ECM around neuronal cells	N/A	[36,37,38,39,40,41]
EphrinB–EphB (Receptors)	EphrinB–EphB signalling. Astrocytes, IL-1β and TNF-α.	N/A	[42,43,44,45,46]
RAP-103 (Receptors)	RAP-103-CCR2/CCR5 signalling. IL-1β and TNF-α	N/A	[47,48,49,50]
Gut Microbiota	lipopolysaccharides (LPS), macrophages, IL-1β	N/A	[51,52,53,54,55,56]
Spinal cord stimulation (SCS)	Activation of opioid receptors, reduced sensitivity of hyperexcited neurons	Infections at stimulation sites (rare)	[57,58,59,60,61]
Acupuncture	Purinergic signalling	Bleeding and bruises at insertion sites	[62,63,64]
Exercise	IL-1β, TNF-α, IL-6, IL-10; NO synthesis	N/A	[65,66,67,68,69,70,71,72]

* FDA-approved medication for treating DNP (existing). NOTE: all others are potential treatments/targets.

## References

[1] Feldman E.L., Nave K.A., Jensen T.S., Bennett D.L.H. (2017). New horizons in diabetic neuropathy: Mechanisms, bioenergetics, and pain. Neuron.

[2] Calcutt N.A. (2020). Diabetic neuropathy and neuropathic pain: A (con)fusion of pathogenic mechanisms?. Pain.

[3] Jensen T.S., Karlsson P., Gylfadottir S.S., Andersen S.T., Bennett D.L., Tankisi H., Finnerup N.B., Terkelsen A.J., Khan K., Themistocleous A.C. (2021). Painful and non-painful diabetic neuropathy, diagnostic challenges and implications for future management. Brain.

[4] Feldman E.L., Callaghan B.C., Pop-Busui R., Zochodne D.W., Wright D.E., Bennett D.L., Bril V., Russell J.W., Viswanathan V. (2019). Diabetic neuropathy. Nat. Rev. Dis. Primers.

[5] Oates P.J. (2008). Aldose reductase, still a compelling target for diabetic neuropathy. Curr. Drug Targets.

[6] Singh R., Kishore L., Kaur N. (2014). Diabetic peripheral neuropathy: Current perspective and future directions. Pharmacol. Res..

[7] Lukic I.K., Humpert P.M., Nawroth P.P., Bierhaus A. (2008). The RAGE pathway: Activation and perpetuation in the pathogenesis of diabetic neuropathy. Ann. N. Y. Acad. Sci..

[8] Pop-Busui R., Ang L., Holmes C., Gallagher K., Feldman E.L. (2016). Inflammation as a Therapeutic Target for Diabetic Neuropathies. Curr. Diab. Rep..

[9] Mora C., Navarro J.F. (2006). Inflammation and diabetic nephropathy. Curr. Diabetes Rep..

[10] Gosselin R.D., Suter M.R., Ji R.R., Decosterd I. (2010). Glial cells and chronic pain. Neuroscientist.

[11] Zenker J., Ziegler D., Chrast R. (2013). Novel pathogenic pathways in diabetic neuropathy. Trends Neurosci..

[12] Edwards J.L., Vincent A.M., Cheng H.T., Feldman E.L. (2008). Diabetic neuropathy: Mechanisms to management. Pharmacol. Ther..

[13] Pittenger G., Vinik A. (2003). Nerve growth factor and diabetic neuropathy. Exp. Diabesity Res..

[14] Tomlinson D.R., Fernyhough P., Diemel L.T. (1997). Role of neurotrophins in diabetic neuropathy and treatment with nerve growth factors. Diabetes.

[15] Obrosova I.G., Li F., Abatan O.I., Forsell M.A., Komjáti K., Pacher P., Szabó C., Stevens M.J. (2004). Role of poly(ADP-ribose) polymerase activation in diabetic neuropathy. Diabetes.

[16] Elliott J., Sloan G., Stevens L., Selvarajah D., Cruccu G., Gandhi R.A., Kempler P., Fuller J.H., Chaturvedi N., Tesfaye S. (2023). Female sex is a risk factor for painful diabetic peripheral neuropathy: The EURODIAB prospective diabetes complications study. Diabetologia.

[17] Singleton J.R., Smith A.G. (2012). The diabetic neuropathies: Practical and rational therapy. Semin. Neurol..

[18] Qureshi Z., Ali M.N., Khalid M. (2022). An Insight into Potential Pharmacotherapeutic Agents for Painful Diabetic Neuropathy. J. Diabetes Res..

[19] Alshehri F.S. (2023). Tapentadol: A Review of Experimental Pharmacology Studies, Clinical Trials, and Recent Findings. Drug Des. Dev. Ther..

[20] Moore R.A., Straube S., Wiffen P.J., Derry S., McQuay H.J. (2009). Pregabalin for acute and chronic pain in adults. Cochrane Database Syst. Rev..

[21] Knadler M.P., Lobo E., Chappell J., Bergstrom R. (2011). Duloxetine: Clinical pharmacokinetics and drug interactions. Clin. Pharmacokinet..

[22] Anand P., Privitera R., Donatien P., Fadavi H., Tesfaye S., Bravis V., Misra V.P. (2022). Reversing painful and non-painful diabetic neuropathy with the capsaicin 8% patch: Clinical evidence for pain relief and restoration of function via nerve fiber regeneration. Front. Neurol..

[23] Tesfaye S., Tesfaye S., Sloan G., Sloan G., Petrie J., Petrie J., White D., White D., Bradburn M., Bradburn M. (2022). Comparison of amitriptyline supplemented with pregabalin, pregabalin supplemented with amitriptyline, and duloxetine supplemented with pregabalin for the treatment of diabetic peripheral neuropathic pain (OPTION-DM): A multicentre, double-blind, randomised crossover trial. Lancet.

[24] Bouhassira D., Tesfaye S., Sarkar A., Soisalon-Soininen S., Stemper B., Baron R. (2023). Efficacy and safety of eliapixant in diabetic neuropathic pain and prediction of placebo responders with an exploratory novel algorithm: Results from the randomized controlled phase 2a PUCCINI study. Pain.

[25] Faheem M., Khan A.U., Shah F.A., Li S. (2022). Investigation of Natural Compounds for Therapeutic Potential in Streptozotocin-induced Diabetic Neuroinflammation and Neuropathic Pain. Front. Pharmacol..

[26] Chen P., Song X.J. (2023). Vitamins in neuropathy: Pathophysiological and therapeutic roles. Curr. Opin. Neurol..

[27] Karaganis S., Song X.J. (2021). B vitamins as a treatment for diabetic pain and neuropathy. J. Clin. Pharm. Ther..

[28] Mäder R., Deutsch H., Siebert G.K., Gerbershagen H.U., Grühn E., Behl M., Kübler W. (1988). Vitamin status of inpatients with chronic cephalgia and dysfunction pain syndrome and effects of a vitamin supplementation. Int. J. Vitam. Nutr. Res..

[29] Stracke H., Lindemann A., Federlin K. (1996). A benfotiamine-vitamin B combination in treatment of diabetic polyneuropathy. Exp. Clin. Endocrinol. Diabetes.

[30] Yxfeldt A., Wallberg-Jonsson S., Hultdin J., Rantapää-Dahlqvist S. (2003). Homocysteine in patients with rheumatoid arthritis in relation to inflammation and B-vitamin treatment. Acta Rheumatol. Scand..

[31] Wang Z.B., Gan Q., Rupert R.L., Zeng Y.M., Song X.J. (2005). Thiamine, pyridoxine, cyanocobalamin and their combination inhibit thermal, but not mechanical hyperalgesia in rats with primary sensory neuron injury. Pain.

[32] Song X.S., Huang Z.J., Song X.J. (2009). Thiamine suppresses thermal hyperalgesia, inhibits hyperexcitability, and lessens alterations of sodium currents in injured, dorsal root ganglion neurons in rats. Anesthesiology.

[33] He D.D., Gao Y., Wang S., Xie Z., Song X.J. (2020). Systematic Administration of B Vitamins Alleviates Diabetic Pain and Inhibits Associated Expression of P2 × 3 and TRPV1 in Dorsal Root Ganglion Neurons and Proinflammatory Cytokines in Spinal Cord in Rats. Pain Res. Manag..

[34] Sharma P., Rani N., Gangwar A., Singh R., Kaur R., Upadhyaya K. (2023). Diabetic Neuropathy: A Repercussion of Vitamin D Deficiency. Curr. Diabetes Rev..

[35] El-Sawaf E.S., Saleh S., Abdallah D.M., Ahmed K.A., El-Abhar H.S. (2021). Vitamin D and rosuvastatin obliterate peripheral neuropathy in a type-2 diabetes model through modulating Notch1, Wnt-10α, TGF-β and NRF-1 crosstalk. Life Sci..

[36] Escolano-Lozano F., Gries E., Schlereth T., Dimova V., Baka P., Vlckova E., König S., Birklein F. (2021). Local and Systemic Expression Pattern of MMP-2 and MMP-9 in Complex Regional Pain Syndrome. J. Pain.

[37] Singh P., Bansal S., Kuhad A., Kumar A., Chopra K. (2020). Naringenin ameliorates diabetic neuropathic pain by modulation of oxidative-nitrosative stress, cytokines and MMP-9 levels. Food Funct..

[38] Kawasaki Y., Xu Z.Z., Wang X., Park J.Y., Zhuang Z.Y., Tan P.H., Gao Y.J., Roy K., Corfas G., Lo E.H. (2008). Distinct roles of matrix metalloproteases in the early- and late-phase development of neuropathic pain. Nat. Med..

[39] Liu W.T., Han Y., Liu Y.P., Song A.A., Barnes B., Song X.J. (2010). Spinal matrix metalloproteinase-9 contributes to physical dependence on morphine in mice. J. Neurosci..

[40] Deng X., Ma P., Wu M., Liao H., Song X.J. (2021). Role of Matrix Metalloproteinases in Myelin Abnormalities and Mechanical Allodynia in Rodents with Diabetic Neuropathy. Aging Dis..

[41] Lu J., Yang L., Xu Y., Ai L., Chen J., Xiong F., Hu L., Chen H., Liu J., Yan X. (2021). The Modulatory Effect of Motor Cortex Astrocytes on Diabetic Neuropathic Pain. J. Neurosci..

[42] Song X.J., Cao J.L., Li H.C., Zheng J.H., Song X.S., Xiong L.Z. (2008). Upregulation and redistribution of ephrinB and EphB receptor in dorsal root ganglion and spinal dorsal horn neurons after peripheral nerve injury and dorsal rhizotomy. Eur. J. Pain.

[43] Song X.J., Zheng J.H., Cao J.L., Liu W.T., Song X.S., Huang Z.J. (2008). EphrinB-EphB receptor signaling contributes to neuropathic pain by regulating neural excitability and spinal synaptic plasticity in rats. Pain.

[44] Liu S., Liu W.T., Liu Y.P., Dong H.L., Henkemeyer M., Xiong L.Z., Song X.J. (2011). Blocking EphB1 receptor forward signaling in spinal cord relieves bone cancer pain and rescues analgesic effect of morphine treatment in rodents. Cancer Res..

[45] Liu S., Liu Y.P., Song W.B., Song X.J. (2013). EphrinB-EphB receptor signaling contributes to bone cancer pain via Toll-like receptor and proinflammatory cytokines in rat spinal cord. Pain.

[46] Deng X., Wu M., Xu N., Ma P., Song X.J. (2017). Activation of ephrinB-EphB receptor signalling in rat spinal cord contributes to maintenance of diabetic neuropathic pain. Eur. J. Pain.

[47] Ruff M.R., Inan S., Shi X.Q., Meissler J.J., Adler M.W., Eisenstein T.K., Zhang J. (2022). Potentiation of morphine antinociception and inhibition of diabetic neuropathic pain by the multi-chemokine receptor antagonist peptide RAP-103. Life Sci..

[48] White F.A., Feldman P., Miller R.J. (2009). Chemokine signaling and the management of neuropathic pain. Mol. Interv..

[49] Padi S.S.V., Shi X.Q., Zhao Y.Q., Ruff M.R., Baichoo N., Pert C.B., Zhang J. (2012). Attenuation of rodent neuropathic pain by an orally active peptide, RAP-103, which potently blocks CCR2- and CCR5-mediated monocyte chemotaxis and inflammation. Pain.

[50] Munawar N., Bitar M.S., Masocha W. (2023). Activation of 5-HT1A Receptors Normalizes the Overexpression of Presynaptic 5-HT1A Receptors and Alleviates Diabetic Neuropathic Pain. Int. J. Mol. Sci..

[51] Fan Y., Pedersen O. (2021). Gut microbiota in human metabolic health and disease. Nat. Rev. Microbiol..

[52] Shen S., Lim G., You Z., Ding W., Huang P., Ran C., Doheny J., Caravan P., Tate S., Hu K. (2017). Gut microbiota is critical for the induction of chemotherapy-induced pain. Nat. Neurosci..

[53] Lin B., Wang Y., Zhang P., Yuan Y., Zhang Y., Chen G. (2020). Gut microbiota regulates neuropathic pain: Potential mechanisms and therapeutic strategy. J. Headache Pain.

[54] Delprete C., Rimondini Giorgini R., Lucarini E., Bastiaanssen T.F.S., Scicchitano D., Interino N., Formaggio F., Uhlig F., Ghelardini C., Hyland N. (2023). Disruption of the microbiota-gut-brain axis is a defining characteristic of the α-Gal A (-/0) mouse model of Fabry disease. Gut Microbes.

[55] Guo R., Chen L.H., Xing C., Liu T. (2019). Pain regulation by gut microbiota: Molecular mechanisms and therapeutic potential. Br. J. Anaesth..

[56] Ma P., Mo R., Liao H., Qiu C., Wu G., Yang C., Zhang Y., Zhao Y., Song X.J. (2022). Gut microbiota depletion by antibiotics ameliorates somatic neuropathic pain induced by nerve injury, chemotherapy, and diabetes in mice. J. Neuroinflamm..

[57] Liampas A., Rekatsina M., Vadalouca A., Paladini A., Varrassi G., Zis P. (2020). Non-Pharmacological Management of Painful Peripheral Neuropathies: A Systematic Review. Adv. Ther..

[58] Sato K.L., King E.W., Johanek L.M., Sluka K.A. (2013). Spinal cord stimulation reduces hypersensitivity through activation of opioid receptors in a frequency-dependent manner. Eur. J. Pain.

[59] Sdrulla D., Guan Y., Raja S.N. (2018). Spinal cord stimulation: Clinical efficacy and potential mechanisms. Pain Pract..

[60] Slangen R., Schaper N.C., Faber C.G., Joosten E.A., Dirksen C.D., van Dongen R.T., Kessels A.G., van Kleef M. (2014). Spinal cord stimulation and pain relief in painful diabetic peripheral neuropathy: A prospective two-center randomized controlled trial. Diabetes Care.

[61] de Vos C.C., Meier K., Zaalberg P.B., Nijhuis H.J., Duyvendak W., Vesper J., Enggaard T.P., Lenders M.W. (2014). Spinal cord stimulation in patients with painful diabetic neuropathy: A multicentre randomized clinical trial. Pain.

[62] Tang Y., Yin H.Y., Rubini P., Illes P. (2016). Acupuncture-Induced Analgesia: A Neurobiological Basis in Purinergic Signaling. Neuroscientist.

[63] Garrow A.P., Xing M., Vere J., Verrall B., Wang L., Jude E.B. (2014). Role of acupuncture in the management of diabetic painful neuropathy (DPN): A pilot Rct. Acupunct. Med..

[64] Qu S.Y., Wang H.Z., Hu Q.Q., Ma Y.Q., Kang Y.R., Ma L.Q., Li X., Chen L.H., Shao X.M., Liu B.Y. (2023). Electroacupuncture may alleviate diabetic neuropathic pain by inhibiting the microglia P2 × 4R and neuroinflammation. Purinergic Signal..

[65] Luo J., Zhu H.Q., Gou B., Zheng Y.L. (2022). Mechanisms of exercise for diabetic neuropathic pain. Front. Aging Neurosci..

[66] Ma X.Q., Qin J., Li H.Y., Yan X.L., Zhao Y., Zhang L.J. (2019). Role of exercise activity in alleviating neuropathic pain in diabetes via inhibition of the pro-inflammatory signal pathway. Biol. Res. Nurs..

[67] Nadi M., Bambaeichi E., Marandi S.M. (2019). Comparison of the effect of twotherapeutic exercises on the inflammatory andphysiological conditions and complications of diabetic neuropathy in female patients. Diabetes Metab. Syndr. Obes..

[68] Yoo M., D’Silva L.J., Martin K., Sharma N.K., Pasnoor M., LeMaster J.W., Kluding P.M. (2015). Pilot study of exercise therapy on painful diabetic peripheral neuropathy. Pain Med..

[69] Jensen T.M., Eriksen SB M., Larsen J.S., Aadahl M., Rasmussen S.S., Olesen L.B., Rehling T., Molsted S. (2019). Exercise training is associated with reduced pains from the musculoskeletal system in patients with type 2 diabetes. Diabet. Res. Clin. Pract..

[70] Heidari M., Zolaktaf V., Ghasemi G., Nejadian S.L. (2021). Integrated exercise glycemic peripheral sensation control in diabetic neuropathy: A single-blind randomized controlled trial. Int. J. Prev. Med..

[71] Olver T.D., McDonald M.W., Grisé K.N., Dey A., Allen M.D., Medeiros P.J., Lacefield J.C., Jackson D.N., Rice C.L., Melling C.W.J. (2014). Exercise training enhances insulin-stimulated nerve arterial vasodilation in rats with insulin-treated experimental diabetes. Am. J. Physiol. Regul. Integr. Comp. Physiol..

[72] Nascimento P.S., Lovatel G.A., Ilha J., Xavier L.L., Schaan B.D., Achaval M. (2012). Exercise alleviates hypoalgesia and increases the level of calcitonin gene-related peptide in the dorsal horn of the spinal cord of diabetic rats. Clinics.

[73] Bhandari R., Sharma A., Kuhad A. (2022). Novel Nanotechnological Approaches for Targeting Dorsal Root Ganglion (DRG) in Mitigating Diabetic Neuropathic Pain (DNP). Front. Endocrinol..

[74] Eissa R.G., Eissa N.G., Eissa R.A., Diab N.H., Abdelshafi N.A., Shaheen M.A., Elsabahy M., Hammad S.K. (2023). Oral proniosomal amitriptyline liraglutide for management of diabetic neuropathy: Exceptional control over hyperglycemia neuropathic pain. Int. J. Pharm..

